# Extracts

**Published:** 1883-08

**Authors:** 


### ATLANTA MEDICAL REGISTER.

VOL. II.] AUGUST, 1883. [No. 11

### Extracts.

LEPROSY IN THE UNITED STATES.—(*Henry G. Piffard, M.D., in Journal of Cutaneous and Venereal Diseases*).—The increasing prevalence of leprosy in this country, and the admitted fact that it is capable of being transferred to previously healthy persons, renders it expedient that special attention should be called to it at this time. The writer's first experience with the affection dates back to the year 1864, at which time he served as interne at Bellevue Hospital, in one of the wards of which there was a leper. Since then there has come under his observation upwards of thirty cases. Some of these were under his immediate care, while others were either private or hospital patients of his professional friends. This experience has enabled him to observe the disease in all of its principal forms, both pure and complicated with other affections. It, however, is dwarfed into insignificance in comparison with that obtained by many observers in parts of the world where the disease is endemic, or has acquired a decided foot-hold. At the present time the writer has under his care four cases in the dermatological wards of the Charity Hospital, on Blackwell's Island, New York. The history of these cases will be briefly given, after which there will be a few remarks concerning the disease in general, especially from the stand-points of diagnosis, prophylaxis, and treatment.

*Case I.*—James E., now twenty-two years of age, consulted me concerning an eruption on the skin about two and a half years ago. On inquiry I obtained the following history: He was born on the Island of Bermuda, of English parentage, suffered from typhoid and yellow fevers, and the usual diseases of childhood, and was brought to this country by his parents when about twelve years of age. After residing here about two years he noticed brownish discolorations about the hips, followed by similar lesions on the thighs, legs and arms. A year later he returned to Bermuda, where the affection gradually became worse, and tubercular bosses appeared on the forehead. These later appeared on the legs and arms. He returned to the United States about three years ago, and shortly after came under the writer's observation. At that time he exhibited the tubercular form of the disease in its most characteristic phase. In addition there were evidences of the previous macular condition, as well as impairment of sensation in the extremities. I advised him to enter the Charity Hospital, which advice he followed, and he has been there ever since.

*Case II.*—Peter A. first came under notice in January last, bearing a letter from Dr. M. Magelssen, of Albert Lea, Minnesota, who had referred him to me. From Peter, I obtained the following history: He was twenty-six years of age and was born in Norway. He came to the United States when a child, and has resided here ever since. Beside the other members of his family who immigrated at the same time, there was an aunt who was a sufferer from leprosy. Six years ago the first symptoms of his present disease appeared. When he came under notice his face and body were covered with tubercular and ulcerative lesions, and there was impairment of sensation at different parts of the cutaneous surface. He was admitted into Charity Hospital January 23, 1883.

*Case III.*—Adolph M., a native of the West Indies, was admitted into Charity Hospital March 14, 1883. He states that his father was a Dane and his mother a native of the West Indies. About eleven months ago sores appeared on various parts of his body, together with small lumps on his face. He came under personal observation April 1, at which time the following lesions were present: First, pustulo-crustaceous lesions having the aspect of syphilitic rupia; second, a profuse tubercular eruption involving the face and ears; third, macular lesions on various parts; fourth, enlargement of the ulnar nerves; fifth, impairment of sensation. The diagnosis was leprosy and syphilis. He was placed on anti-syphilitic treatment, and in a few weeks the pustulo-crustaceous lesions disappeared, while the other lesions mentioned were unaffected by the treatment. During the month of May he had an attack of leprous fever, followed by an eruption of characteristic leprous tubercles on the leg.

*Case IV.*—Oney J., born in China, twenty-four years of age, was admitted into Charity Hospital April 2, 1883. This patient came to the United States when quite young, residing the greater part of the time in California. His present trouble first made its appearance three and a half years ago, as a tubercular eruption on both cheeks, which gradually extended over the face. On admission his face was found to be covered with tubercular masses, varying from the size of a pea to that of an almond. On the back and elsewhere there were leprous discolorations, the ulnar nerves were enlarged, and there was hyperæsthesia of the skin of the extremities.

*History.*—From the earliest times of which history gives account, we find mention of the disease now commonly known by the name of leprosy. The word occurs in the English versions of both the Old and New Testaments, and in the former undoubtedly has reference to the disease under present consideration. In the New Testament the word appears as a translation of the Greek word *aenpa*, a term applied by the older Greek medical writers to the disease now called Psoriasis. Leprosy proper was called Elephantiasis, to which name modern writers usually add the term *Grœcorum*, to distinguish the affection from a totally different disease called elephantiasis by the Arabians.

Leprosy has prevailed to a greater or less degree in almost every portion of the known world, from the frigid zone to the tropics; but at the present time is chiefly found under the extremes of temperature, being much less prevalent in the temperate zones. In North America it prevails in Mexico, Louisiana, California (among the Chinese), Wisconsin and Minnesota (among Norwegian settlers), in New Brunswick, B. A., and among the Indians of the Northwest. Besides this, cases are encountered among immigrants from countries where the disease is specially prevalent, particularly the West Indies, South America and the Sandwich Islands. It is from the three latter countries that most of the cases have come that have fallen under my observation.

*Lesions, Varieties, and Symptomatology.*—The lesions of leprosy occur in the nervous system, in the mucous membranes, in the skin, and elsewhere. In the nervous system we find changes in the spinal cord and nerves, the ulnar nerve being the one most conspicuously affected. Among the earlier changes are swelling of the nerve, followed by atrophy. The mucous membranes, particularly of the mouth and throat, become the seat of tubercular formations, which, later, may undergo ulceration. On the skin we find macules, bullæ, tubercles and ulcerations. The three principal varieties or types of the disease are the macular, the anæsthetic, and the tubercular. It is exceedingly rare to find an individual case, however, that exhibits either type in its purity. As a rule, all cases exhibit a mixture of types, with one or the other prevailing. Before the symptoms of the disease become clear and unmistakable, there is usually a prodromal stage of varying duration, characterized simply by a gradual impairment of the general powers, both physical and mental. Occasionally a brownish discoloration (*macule*), or an isolated bulla may appear from time to time, the first one usually healing before the second one makes its appearance. Later the macules become more abundant and larger, reaching the size of the palm of the hand, but are not accompanied with any appreciable degree of infiltration. At first they are of a reddish-brown, and as they increase peripherally, their advancing border retains this color, while the more central portions lose it, and fade away into a dirty gray, or even a dead white. The sites of these discolorations are at first slightly hyperæsthetic, but as the disease progresses, this condition gradually disappears and is succeeded by anæsthesia. In connection with the macules, or even without them, tubercles may arise. These are thickened elevations of the skin, sometimes quite circumscribed, at other times more diffuse. The tubercles may exhibit the normal coloration of the skin, or the color may be heightened. At first they may be hyperæsthetic, later becoming anæsthetic. They may appear upon any part of the body, but usually make the face their favorite seat, showing themselves on the forehead, about the lips, and on the ears. When they are developed to any great extent, they render the features repulsive and disgusting to the last degree, sometimes causing a leonine appearance. The tubercles frequently persist unchanged for a very long time, even perhaps throughout the whole continuance of the disease. Sometimes, however, they undergo ulceration, or disappear by interstitial absorption. Accompanying the tubercles there may be patches of skin which are anæsthetic, but which exhibit no other apparent change. This anæsthesia may be temporary or permanent. Coincidentally, or subsequently, the mucous membranes of the buccal cavity, nares, pharynx, etc., may exhibit tubercular lesions.

The *anæsthetic* form of leprosy may arise as a late stage in the course of a case which, at the beginning, had exhibited macular or tubercular features only, or it may arise without such previous tubercular development. The cutaneous lesions met with at the beginning of this form are usually *bullæ*, which vary in size and persist for a short time only. They usually rupture, dry up, and leave a stain, which after a time becomes anæsthetic. Hyperæsthetic patches of varying extent may appear from time to time, and persist for months or longer, and be ultimately succeeded by anæsthesia. The finger ends often become enlarged and clubbed, and covered with glossy skin. The anæsthetic portions of skin may undergo a certain degree of atrophy, which processes may also involve the subcutaneous tissues, and result in ulceration, and if situated on the hands or feet, to caries of the bones of these parts. This may result in total loss of the fingers and toes, and even involvement of the metacarpal and metatarsal bones.

According to the researches of Danielsen and Boeck, in Norway, the average duration of the disease is about twelve to fifteen years.

*Diagnosis.*—The diagnosis of leprosy in the very earliest stages is unquestionably difficult, and in many cases undoubtedly impossible; but when the disease has fairly opened, and exhibits any of the lesions mentioned, namely, brownish patches, bullæ, tubercles, sensory disturbances, enlargement of the ulnar nerves, etc., suspicion should be awakened, and if on inquiry it be ascertained that the patient has formerly lived in a country where the disease is endemic, or has been in familiar intercourse with a person suffering from leprosy, the suspicion will be greatly strengthened. If two or more of the symptoms mentioned are found to be present, the diagnosis is almost certain. As regards differential diagnosis, the following points should be borne in mind: sensory disturbances should be differentiated from those that result from other essential affections of the spinal cord and nervous trunks; bullæ from those dependent on pemphigus; tubercles from the similar lesions of syphilis. While in the great majority of cases the diagnosis can be made with little difficulty when the lesions are well pronounced, still occasionally it is otherwise. The writer some time since was asked to see a case in which he was unable to decide whether the patient had leprosy or syphilis, or both, and there is in the city at present a case in which there is doubt as to whether it be a case of leprosy or some essential disease of the spinal cord.

*Etiology.*—The etiology of leprosy is obscure. Diet, climatic influence, and bad hygiene, all have been brought forward to account for the prevalence of the disease in certain countries. These, however, cannot be regarded as entitled to much weight, inasmuch as we find the most opposite conditions prevailing in this respect in regions where the disease is of frequent occurrence. Hereditary influence is a much more probable factor. Direct contagion, however, is unquestionably the most frequent cause of the spread of the disease, though the exact manner in which it is effective has not yet been discovered. This question, however, has been so ably discussed by Dr. J. C. White in a paper read before the American Dermatological Association, that we will simply refer the reader to that paper, or to the abstract of it that appears among the selected articles in the present number of this *Journal.*

*Histology.*—During the past few years the histology of leprosy has undergone a complete revision, but cannot as yet be considered as fully established. The changes in the nervous system have been carefully studied by Hoggan and others, while the discovery by Hansen and Neisser of bacilli in the tubercles throw new light on the subject, but the exact bearing of these researches cannot as yet be considered as definitely settled.

*Prophylaxis.*—We presume that no one will dispute the proposition that it would be exceedingly unfortunate if leprosy should gain the foothold among us that it has acquired among certain other peoples, the Norwegians, for instance, and still more notably, among the inhabitants of the Sandwich Islands. It is hardly more than forty years since it was first introduced among the inhabitants of those islands; yet in that time it has already claimed nearly ten per cent. among the natives of that region as victims. It is not at all probable that the disease would spread in America with anything like the rapidity that it has in some other countries, but the fact remains that several cases of leprosy are on record in which the disease originated in this country under circumstances that lead directly to the conclusion that it is perfectly capable of increasing among us from direct contact with imported lepers, as in the case of Peter A. reported above. Under these circumstances, it would seem wise that measures should be taken early, while the evil is still in its infancy, to protect the people from further danger.

This can be accomplished only through national legislation; and to this end we would urge on Congress the necessity of providing, first, a central Lazaretto; second, immuring therein all lepers now in this country; and third, watching immigration, and giving each leprous immigrant the option of returning to the country whence he came, or of entering the Lazaretto. This will have to be done sooner or later, and the sooner it is done, the less difficult will be the undertaking.

*Treatment.*—As regards this branch of the subject, it may be briefly stated that, as yet, no specific for the disease has been discovered. It is also problematical whether the affection is susceptible of absolute cure. Per contra, we are fully justified in stating that rapid amelioration may often follow judicious treatment, even in the advanced stages of the disease, as we have more than once witnessed. We have recently (*Materia Medica and Therapeutics of the Skin*, pp. 200-208) considered this subject at length, and can at present state that the drugs that have appeared to exert the most decided influence on the affection are nux vomica and its derivatives, and chaulmoogra oil. With these, the progress of the disease can be stayed, at least for a time, and the unhappy sufferers restored to a condition of comparative comfort. More than this we do not feel warranted in promising.

---

Clinical Lecture on Some of the Causes of Sterility, and their Treatment.—(*T. Gaillard Thomas, M.D., in Boston Med. and Surg. Journal*).—Three cases of sterility having presented themselves by accident at the clinic to day, I will avail myself of the opportunity to give you a practical illustration of some of the causes and of the treatment of this condition. If your experience when you are in practice is anything like mine has been, however, you will undoubtedly find that in general the results of the treatment of sterility are very unsatisfactory. Perhaps out of twenty cases you may have one or two successes, and your professional neighbor may be led to form a high opinion of your skill, because you will be sure to publish the one or two successes, but will take good care not to say anything about the eighteen or nineteen failures. In a London review of one of the editions of my work on diseases of women, the opinion was expressed that it showed an extraordinary barrenness of results in the treatment of sterility, and that these were much better on the other side of the water. Yet if one were to examine into the methods resorted to there it would be found that precisely the same means were employed which were recommended in my book.

There is only one way in which any success whatever can be achieved in the treatment of this troublesome affection, and that is to study each particular case on its own merits, and thus avoid the grievous error of falling into any routine method.

In doing this one will sometimes find that there is some constitutional ailment in the husband which prevents conception; in other cases, that although there is no constitutional trouble, the local results of a specific urethritis give rise to the difficulty, and in others, again, that the husband is impotent.

The first patient that I bring before you is Mrs. J., who is twenty seven years of age, and has been married eight years, but has never been pregnant, although she is anxious to have children. She says that she enjoys good health, with the exception of having more or less pain in her head and back, and marked irregularity in her monthly sickness. Her menses come on only once in every three or four months, and the flow even then is very scanty, at the most lasting only twenty-four hours; while it is accompanied by very severe bearing-down pain. She also suffers considerably from leucorrhœa.

From the above history we can say at the outset that it looks as if there were something wrong about the woman herself, rather than her husband, which prevents her from bearing children. The results of a physical examination are as follows: The patient having been placed on the back and the finger passed up into the vigina, it was found that the cervix was in its normal position, but the moment it was touched it was ascertained that there was something peculiar about it. The os, instead of being of the natural size, was only that of a small pin-hole. The finger was now carried up higher and a mass was found behind the cervix pressing upon the rectum. By conjoined manipulation it was ascertained that the body of the uterus was absent from its natural position, and the patient then being placed upon her side and a Sims speculum introduced, a very small probe, bent backward, was passed down into the uterine cavity. It was thus demonstrated beyond a question that the mass back of the cervix was the body of the uterus, which was in a state of marked retroflexion. We have found, therefore, an ample explanation of the sterility in this case. It is true that many women suffering from retroflexion do bear children (though they generally have one or two miscarriages first), but when this is the case, you will not find the os in the condition which we have noted here.

In answer to special inquiries, the patient informs me that she did not begin to menstruate until she was eighteen years of age, and that she had to have the mouth of the womb opened by a slight operation. In these cases of narrow cervix the blood gets dammed up, causing great pain, which is at length relieved by the expulsion of a small clot. After a time, as the result of this repeated damming up of the blood in the uterine cavity, the organ becomes retroflexed. We then have an additional factor in the production of dysmenorrhœa, because the bending of the uterus gives rise to spasmodic spasm of the sphincter muscular fibres of the cervix, and spasmodic dysmenorrhœa is thus added to the obstruction. We have, therefore, three causes for the trouble in this case: First, a narrow cervix, preventing a free egress of menstrual blood, and causing a "ballooning" of the uterus at every period; second, retroflexion, occasioning spasm of the cervical muscle; and, third, a constant engorgement of the uterus with blood, resulting in the production of uterine catarrh.

Before referring to the treatment appropriate here, I would warn you never to promise a cure in such a case as this. Simply tell your patient that you see a number of conditions which appear to prevent pregnancy, that you think you can remove them, and that when this has been done the chances are that her dysmenorrhœa will be relieved, and that she will bear children. The treatment must consist in overcoming the chain of morbid conditions discovered, the narrow cervix, the bent uterus, the uterine spasm, and the uterine catarrh. It would be better, however, not to commence their removal in in the order in which they have occurred. Such a patient should enter the hospital, and, having been put to bed, should be treated, first of all, with the hot water douche and laxatives. Then three times a week the uterus should be restored to its normal position by means of the sound. It is probable that her next menstrual period would be less painful than heretofore, and at the end of it the cervix should be held with a tenaculum, and three incisions, each cutting the sphincter muscle to the depth of one-eighth of an inch, should be made with the Sims knife. This having been done thoroughly, a glass stem should be pushed up into the uterus and maintained in position by means of an Albert Smith pessary (provided with a little cup in which the lower extremity of the stem can rest), which will also keep the uterus in position. After the operation the patient should be placed in bed and given an opiate. She should remain in bed for ten or twelve days, and have a carbolized vaginal injection three times a day. The glass stem may be kept in the uterus until after the next menstrual period if it is thought desirable, but it should at all events be taken out then. By this time it will probably be found that the uterine engorgement has disappeared and the leucorrhœa has diminished; while the unnatural narrowness of the cervix has been removed entirely. (The glass stem should be somewhat larger than the normal canal.)

The patient can then be allowed to go home, wearing the stem and Albert Smith pessary. Twice a week the stem should be removed, and a probe, wrapped in cotton, dipped in pure carbolized acid passed up to the fundus. This application, which covers the mucous membrane with a whitish coating, can be made in the office. It is accompanied with no danger whatever, and I regard the carbolic acid as of much more service than iodine, zinc, or alum when used in this way. In from six months to a year the glass stem might be left off, and there would then be a chance of impregnation. It would be only a chance, you must remember, but I know of no better plan of treatment than that of which I have now given you the outline. Our premises here, I think, are undoubtedly correct, as I have seen the same conditions in hundreds of cases. In this particular instance, however, I should not feel very hopeful of overcoming the sterility, for the reason that the facts that the patient did not commence to menstruate until she was eighteen, and that the flow only came on every three months, naturally make me suspect that the ovaries may be at fault as well as the uterus.

Our next patient is Mrs. Elizabeth L., born in Germany, and nineteen years old. She has been married four years, and has never had any children, but thinks she had one miscarriage. She complains of pain in the region of both ovaries, which begins four days before the menstrual flow, continues through it, and lasts for a day or two after it disappears. She also suffers from the pain in the top of the head, which has been called uterine headache, and says she has never felt well since she had the miscarriage, which occurred about a year after her marriage.

Each case of sterility must be studied by itself, and this is of an entirely different character from the last. When a vaginal examination was made here the finger failed to reach the cervix in its normal positions, but it was afterwards found much higher up than it ought to have been, while a mass continuous with this was found pressing down upon the rectum. The introduction of the probe showed that this was the retroverted uterus, the axis of the organ being completely changed. From the os uteri could be seen, through the speculum, a plug of viscid mucus hanging, looking like white of egg, and so tenacious that it was difficult to cleanse the cervix. When the mucus was removed, a condition was observed beneath resembling that of granular lids, and the surface bled freely when touched with the small, dry sponge used to cleanse it. Both ovaries were enlarged to twice the normal size.

Here is a young woman who was perfectly well up to the time of a miscarriage, occurring a year after marriage. She very likely got up on the third or fourth day, and in all probability, unfortunately, with the consent of her physician, if she had one. The uterus being heavy, fell over backwards, and the ligaments were pulled upon, the traction on the broad ligaments preventing the return of venous blood. The pathological chain in this case is, then, retroversion with engorgement of the uterus, endometritis, chronic inflammation of the Nabothian follicles, and sympathetic engorgement of the ovaries. It is estimated by Tyler Smith that there are two thousand mucous follicles in the cervix, and it is a question whether chronic inflammation of these is ever cured.

The treatment that I would advise here would be to restore the uterus to its normal position, and put in a retroversion pessary, after which the mucus should be carefully removed from the whole uterine tract two or three times a week, and the surface painted over with pure carbolic acid. Within the os internum this is undoubtedly the safest as well as the best application, and I know of no other chemical which, thus applied, is not liable to produce pelvic cellulitis or peritonitis occasionally. Judging from my past experience, however, I should pronounce this even a more doubtful case than the other, as the sterility depends upon a cause well nigh incurable.

Our last patient to-day is Mrs. Rose G., twenty-four years old, and a native of Austria. She has been married six years, but has never been pregnant, and it is on account of her sterility that she comes to consult us. She is regular in her monthly periods, but sometimes they are accompanied with much more pain than at others. The flow lasts from four to six days. When the finger was introduced into the vagina the cervix was found to be long and conical, and the os very small. There was no leucorrhœa or other abnormal condition. The probe was passed with great difficulty on account of the small size of the os, and I have no doubt that in this case the peculiar formation of the cervix is the cause of the sterility. It is a cause which has been recognized by every writer within the last few years, and I think that our patient can, probably, be entirely cured.

In aggravated cases of this kind, where the neck projects very markedly, and is decidedly conoidal, amputation of the cervix is required, but in the present instance I think the cervix hardly long enough for this, and would advise the operation by bilateral incisions for its enlargement. If this should be done it is highly probable that impregnation would result, but it is by no means a certainty. I have thus been able to present to you some cases illustrating a few of the numerous causes of sterility, and it gives me pleasure to conclude with one in which it is possible to make a little better prognosis than in the other two.

---

MEDICAL EMPIRICISMS, HOMŒOPATHY AND CODES.—(*Romaine J. Curtiss, M.D., Joliet, Ill.*)—The medical profession at large are sufficiently enlightened on the present status of what many are pleased to call the old and new codes, or the New York Code, which is new, and the code of the American Medical Association, which is old.

Dr. Austin Flint, in a sort of running commentary, something as an apologist, unites on the sentiments and parables of the holy text, has sufficiently made the matter of the old code plain and reasonable to those who want to believe; while Dr. Roosa has endeavored to uphold the claims of the new code, and after the manner of the speech of Talleyrand, has successfully covered up by his written articles on the subject, every sensible and real motive which could ever have prompted anybody to invent a code which should indiscriminately mix up the creeds, sciences, pathies and superstitions of medicines.

As we might expect, the persons most nearly allied to the merits of these codes by business and locality, have not hesitated to name the probable fact that the real *animus* of the framers of the new code was business interest, as well as a conviction that there is not difference enough between the methods of empirical medicine and homeœopathy to prevent amicable and satisfactory consultations between the two in cases of sickness.

Now to the careful student of medicine, and the close observer of social events, the present condition of "medicine" and homœopathy relating to codes and actual ethical conduct outside of codes relating to consultations, is not matter of surprise, but has been rather a matter of prophesy; and to understand why this is so, let us define what is meant by empirical or rational medicine, and homœopathy.

In the first place, let us note that the word empiricism is a much abused word among physicians who deny that they are empiricists. The word means experiment, or a conclusion derived from trial. Empiricism is, therefore, a word which covers in its meaning the expressive basis of the foundation of all sciences, even medical science. Empiricisms began with Bacon, and empiricism in science means nothing worse than experimental science or conclusions, or facts derived from the conduct of experiments. Dr. Flint, in his running exegesis on the beatitudes of the old code, uses this word in the sense of quackery. It has no shadow of such meaning, nor can any perversion of the meaning of the word make it cover the ethical or professional conduct of a pretender in medicine. Empiricism is the method by which what is called rational medicine was established, and by which the position of rational medicine was reached at the present day; and empiricism is rational medicine as it is practiced to a very great extent to-day, in the absence of what may be called scientific medicine

Empiricism is not scientific medicine, nor is rational medicine scientific; neither is homœopathy; and these two methods are nothing more than the opposite poles of the very same thing in medical art.

Let us first define rational medicine by special definitions relating to the action of medicines and the symptoms of disease, and then we will be able to formulate the general definition, and understand the relation to homœopathy, and both of these to scientific medicine.

The trial by experiment has resulted in the definite formulæ of drugs to each other, and of the antagonism of drugs to signs and symptoms; and it is by these therapeutical formulæ that medicine is practiced all over the world to-day, and it is by these very same formulæ that homœopathy is practiced. It was never learned by any other method than the empirical method that atropia is a physiological antidote to morphia; or that atropia is a physiological antidote to contraction of the pupils; or that it causes dryness of the throat, or lessens or increases the size of the capillaries; or lessens or increases the vascular tonus; or that it antagonizes weakness of the heart's action; or antagonizes cerebral hyperæmia, or causes congestion of the brain and delirium, all depending upon the dose.

Empiricism also has taught us that morphia is the antagonist of pain. Who ever learned this fact by raising poppies or making an analytical or synthetic chemical examination of morphia, and studying the pathology of pain; that each is, to a certain extent, the antagonist of the other? This knowledge was gained by experiment, and was learned before anybody ever knew the chemical synthesis of morphia, or believed that pain was anything but a ghost or an entity.

From centuries of experiment with drugs which were found to antagonize symptoms and signs, people have constructed considerable of a therapeutical system—certainly not a science of medicine.

They have classified symptoms and signs together by association and by likeness and unlikeness, into diseases, and were obliged to do this without reference to the cause or the antecedents, because they couldn't find out the cause or the antecedents, and then from the empirical knowledge the rational physician and the homœopathic physician alike, have proceeded to give the medicines which "experiments" and "provings" have verified to be antagonistic to the signs and symptoms, but without reference to the antagonism of the drug to the antecedents of all the signs and symptoms, which in the aggregate are called a special disease.

Now, so far as we have gone, both rational medicine and homœopathy stand on precisely the same ground. Both treat diseases by giving drugs which antagonize symptoms, and if we take modern rational medicine, and modern homœopathy, we will find that they antagonize the same symptoms by opposite methods, or by opposite in opposite doses, but on equally correct experimental principles! but both methods, though equally rational and empirical, are equally unscientific.

There is no medical science in the method of constructing or classifying a disease from signs and symptoms alone. There is no medical science in medicine if the cause is unknown; and to make medicine scientific, the antecedents of the signs and symptoms must be antagonized by medicine rather than the signs and symptoms themselves.

The antagonism of drugs to each other, and to pathological or rather physiological symptoms, or conditions, or function, is perhaps best studied in Prof. Bartholow's Cartwright lectures on the subject. The subject is here fully presented, and, as any one can see, the methods by which these facts of antagonism were known, were the methods of most laborious experimental empiricism. These methods consist in giving a drug to an animal either in small doses or large doses, and then noting the effect on all functions of all organs, from the brain and nerves to the organs of secretion and excretion. It is upon this basis that the treatment of disease is based. If it is found that a symptom of certain disease is a too abundant perspiration, the empirical doctor will give a dose of oxide of zinc, because experiment has proven that these two things will antagonize each other; while the homœopath will give the minimum dose of atropia because his provings of the drug show that sweating and atropia are antagonistic.

I have said that both of these methods are unscientific because they both ignore the cause of the symptoms in their treatment. Of course, if the cause was known the remedy would be something which could antagonize the cause, and this would be scientific medicine, no matter whether the antagonistic drug was efficient in a small dose or a large dose.

But medicine, not being scientific because the factor of cause is unknown, the place of the unknown factor, in both empiricism and homœopathy is supplied by a dogma to give medicine the appearance of being scientific. The logical construction of the human mind is antagonistic to the conception that the destruction or antagonism of the effects of an unknown cause can destroy the cause itself. What possible effect upon the tubercle baccillus could be brought about by the zinc or atropia which prevents the segment night sweats in the disease caused by this parasite?

The two dogmas, representing rational medicine and homœopathy, are of equal significance, but are simply opposite poles of the same dogmatic error. Rational medicine says the reason why drugs cure disease is because "unlike cures like," and the homœopath says: "because like cures like."

To a certain extent these dogmas are true, but they are equally true and equally untrue. They do not cover that ground of a disease which includes the symptoms and signs as sequences of a known cause, but carried to their logical conclusion their dogmatic nature is fully shown, and both systems are forced to admit that they mean by their dogmas that the cure or remedy in one case is something unlike the cause of disease, and in the other case it is something unlike the cause.

In fact, however, these dogmas are inductions from trials with drugs in the antagonism of signs and symptoms, and the attempt to cover the cause of disease, simply makes them ridiculously absurd. Perhaps either of these schools can prescribe drugs which are antagonistic to the signs of an eruption on the skin in scabies, and to the symptoms of itching, which goes with that disease; but how ridiculous do the dogmas of "unlike cures like" and "like cures like" appear in the light of the known cause, and by remedies which relate not to the direct antagonism of signs and symptoms, but to the destruction of the cause; then we hear no more of rational medicine or homœopathy, but the medical treatment of disease becomes scientific.

Rational medicine and homœopathy are each based on the empirical trial of drugs in the antagonism of the segment signs and symptoms of disease. An examination of the action of drugs will fully account to us for the existence of these two schools' practice, and will fully explain why they stand on equal grounds of error and fact relating to the truth as it is in scientific medicine.

The principle of antagonism to each other, and to symptoms implies that two forces are acting more or less in opposition to each other. These forces are constituted by the physiological action of the drug, and whatever may resist the action of the drug, whether it is a physiological or pathological force, or another drug. The new symptom, or drug symptom, or the segment modification of the physiological force, is the resultant of the drug action, resisted by whatever antagonizes it. If a certain dose of strychnia is given, the action of the drug is resisted by the integrity of the spinal cord, and the resultant is a change in the physiological action of the spinal cord, and other organs for instance, perhaps the normal tension of the muscles is increased to a spasmodic contraction.

This illustrates the principle upon which drugs act when given to antagonize symptoms or functions, and upon this basis we can formulate the action of medicine, and show the true relations of rational medicine to homœopathy.

If the integrity of the spinal cord is normal, and a small dose of strychnia is given, end the resultant of the two antagonistic forces may represent what we can conveniently represent by perhaps 10° or 15°. Now suppose a larger dose is given: the resultant may be 45° or 90°. If a still larger and larger dose is given, the resultant goes on around the circle to 125°, 145° and up to 180°, when we have the external effect of strychnia upon the spinal cord.

Now from the mathematical, therapeutical and physiological standpoint, all these resultants, representing different doses of the drug which are above 90°, are opposite to their corresponding resultants below 00°.

The minimum effect of strychnia is to weaken muscular action, while the extreme effect is to cause tetanic spasm, and the grades of action between these two extremes in physiology and pathology will correspond with all the degrees of the circle.

This same law may be illustrated by the effect of atropia upon the capillary circulation. A small dose, giving its minimum resultants, contracts the capillaries. In the largest dose the capillaries are distended. The effects of the opposite doses are opposite to each other.

We will say that hydrate of chloral is an antagonist to strychnia. Then small doses antagonize the small dose of strychnia, and large doses of each are likewise antagonistic.

Morphia is perhaps an antagonist of atropia, and if so a small dose of morphia will antagonize a small dose of atropia, and large doses of each are antagonistic.

From these data we find that a small dose of strychnia and a large dose of chloral have effects that are similar so far as their antagonistic action goes. If a large dose of chloral then weakens the muscles or relaxes spasm, a small dose of strychnia does the same, and if a small dose of chloral will cause spasm or increase muscular power, a large dose of strychnia will also cause spasm.

If a small dose of atropia contracts the capillaries, and morphia is an antidote, or antagonistic to atropia, then a small dose of morphia should dilate the capillaries, and the dilated capillaries, from large doses of atropia, should be contracted by morphia.

From these data we find there are two legitimate methods of empirical practice of medicine, the rational practice, and the so-called homœopathic practice, as they are both practiced at the present time.

It appears that large doses of some drugs accomplish the same resultants that small doses of other drugs will also accomplish, and without doubt one of these methods is just as effectual as the other, in antagonizing symptoms, and they bear very similar relations of truth and error to the scientific medical practice, which we may say is the impending practice of the future.

In the light of these facts, let us for a moment examine the two dogmas of the two empirical schools, "unlike cures like," and "like cures like."

As we know very well, the primitive homœopath had no hesitation in proclaiming his dogma from the housetops, and in fact, both ancient and modern disciples of this method generally prefer a housetop when they proclaim anything relating to medical practice. Even at the present time some homœopaths, and even some physicians who are not homœopaths, have no hesitation in making the claim that small doses of copaiba cure urticaria *because* large doses cause urticaria, etc. Now the converse of this is the dogma of the other empirical school, "unlike cures like," or a large dose of bromide of potassium will contract the blood-vessels in the brain *because* a large dose of quinine cures cerebral hyperæmia.

If there are any two dogmas on earth any more meaningless than these, I have never heard of them.

To illustrate the practical workings of these systems of symptomatic medicine, we will take the disease of consumption, and review the treatment by symptoms.

In consumption there is a morbid growth in the lungs, which both schools call "error of nutrition," symptomatic of this "error" there are cough, night sweats, emaciation, pain, diarrhœa, etc.

The cause of the error of nutrition being unknown, which causes the morbid growth in the lungs, the medical treatment is limited to the symptoms, with the understanding that the cause is unknown and the prognosis is "unfavorable."

Now both schools agree on the subject of nutrition. Both crowd the digestive organs to their highest capacity, with albumens and fats and alcohols.

The chief symptom is cough, and here is a great field for empirical effort. The different doctors use different medicines in different doses, to diminish the reflex irritation which provokes the cough. Large doses of opium are given on one hand and the small ends of many drugs are tried by the homœopath. The night sweats are treated similarily by empirical methods. On one hand a large dose of oxide of zinc or arom. sulph. acid is prescribed, and the homœopath is equally successful with his little pills of atropia.

The other symptoms are treated in the same way. The heat is abstracted by local cold. On one hand empirical medicine prescribes large doses of quinine, which lessens the cell changes which cause heat, and the little end of aconite is given by the other school for the same purpose.

Now these are the true methods of empiricism in the treatment of diseases, the cause of which is unknown, and unmolested by the doctor. How do these physicians, then, "cure" diseases? Manifestly, they do not cure it. If a symptom exists *per se*, a drug which is a physiological antidote to it, may antagonize the symptom out of existence. But symptoms do not exist *per se*. They must always have an antecedent; symptoms and signs are the methods by which a cause of disease destroys life. The heat of fever, the diarrhœa, sweats, ininition, cough, and tissue metamorphosis in the lungs are the methods by which the tubercle bacillus—the cause of consumption—destroys life. If the symptoms or methods of the cause of disease can be antagonized by drugs until the cause itself is dead, the patient recovers; but if not, the termination is different. If the cause is of a character which is not self-limited as is, probably the cause of consumption, no amount of antagonism of symptoms by drugs, whether by the big or little end of drugs, will do any more than possibly prolong the life of the patient.

The method of empirical treatment of the zymotic diseases. as scarlatina, measles, typhoid, etc., is to antagonize the symptoms or methods by which the poison causes death, until the cause itself is dead by the law of the natural limitation of diseases of this character.

The scientific method of the treatment of disease implies that the cause is known and that there is a method known by which the cause of disease can be destroyed, or rather, prevented. The true science of medical treatment is to destroy the cause of disease before the disease is produced, if it can be done, kill the germ outside the body, if possible; but if not, then kill the germ inside the body, is certainly a more efficient method of medical treatment than simply antagonizing the symptoms which are set up by the germ of disease.

We are at present standing on the brink of discoveries which must revolutionize the methods of physicians in the cure of diseases. If the cause of disease can be destroyed even after the symptoms have declared the nature of the cause, then and not until then can it be said that disease is curable and that physicians really cure disease. The indications now are that physicians really cure disease. The indications now are that the brink of the great discovery upon which we are now standing, is that the cause of disease is some form of living thing—that infection and contagion are really *contagium vivum.*

How obvious it is that if the cause of disease is really known, how short, thereafter, will be the life of emperical medicine. There is no such thing known in the treatment of scabies as rational medicine or homœopathy. The symptoms and signs of this disease, the itching and eruption, are not treated at all, but the remedy is addressed to the cause, and balsam of Peru or sulphur is found to cure this disease, because they can destroy the life of *contagium vivum.*

Now, rational medicine, having become really rational, and having left off its old heroic inquisitional torments of fire, blisters, bleeding, and immense doses; and homœopathy having left off its superstitions about perennial *psora*, and infinitisimal elements, the two are seen to stand upon the same therapeutic ground facing each other, one with a little pill of one drug, and the other with a big pill of another drug, which are found to precisely equal each other in therapeutic force, so far as the antagonism of symptoms goes. What else then could be expected than that the barriers which have separated them so long should be broken down. There is no possible ground of ethical or empirical distinction between these two physicians or classes or schools or methods, which should separate them ethically. If a small dose of a drug will antagonize a symptom as well as a large dose of some other drug, then one of these drugs is no less ethical than the other. No matter which of them has the greatest merit of age, experience or knowledge of symptoms or antidotes, if one method is as good as the other, no matter how high the fence is between them, the fence must come down.

In a measure, and for temporary purposes, the New York New Code, therefore, has its basis in the right, All that is needed is to acknowledge the real facts of difference between the two professions, in order to destroy all the artificial ethics which have been built up between the two. So far as symptomatic doctoring goes, homœpathy stands on just as good grounds as any other of the methods of treating symptoms, and any charges which can be brought against it can also be brought against the rational method of practice, whether the charge is ethical or therapeutical, where one has done too little, the other has done too much. This feature is the historical feature. If one has a foolish dogma, so has the other. If one treats disease by antagonizing symptoms, the other also treats disease by antagonizing symptoms. If there is no science in one, then both are unscientific, and in short, whatever merit or demerit belongs to either, also belongs equally to the other.

But the destiny of pathy is to die. The "medicine" of the future is to be *preventive medicine*. The practice of antagonizing symptoms by either pathical method is simply a confession that the cause of the disease is not known, or if known, there is no remedy known. The time must come when the ignorance relating to the destruction of the cause of disease will give way, and when it does there will be no more impirical method—neither the present rational methods, nor homœopathy. They will gradually merge together, and their methods grow old, and their very names be only repeated and remembered by the student of medical history.

When medicine becomes a science by the discovery of the cause of disease, and its preventive, neither one of these schools will ever attempt a cure of disease by the methods of their dogmas. The blatent voice of dogma in medicine will be as "silent as the grave." In the meantime the interests of the schools in empiricism, if they deserve it, will probably be aided by the general adoption of the New York Code.

of introducing medication, and the nervous tissues serve in like manner as an inlet to medicinal solutions, the results being manifested subsequently upon the general organization. Articles administered by the stomach have to undergo as a rule a species of digestion before they can produce their effects, yet there are some classes of medicine which operate immediately on the coats of this organ, and a stimulant or sedative influence is propagated directly through its nerves to the whole organism. It should not be understood from this statement that all medicines that go into the stomach are limited to these modes of action, as it is ascertained that many agents pass from the stomach with their properties unchanged, and are even detected in the different secretions and excretions of the body, modifying to a greater or less extent the parts with which they came in contact on their route. The external or superficial application of medicinal agents produces local or general effects that are very notable, yet more tardy than by other processes, and are only resorted to in compensation of impediments to other more effective means of treatment. Another and diverse end is attained by the class of cutaneous irritants, whose influence belongs to the important sphere of revulsions, and depends upon the sympathies of the nervous distribution for the striking results.

[To be continued.]

---

A Furious Anti-Vivisectionist.—M. Brown-Sequard was interrupted rather brusquely the other day in one of his courses of experimental physiology at the College de France. A monkey was brought on the table and the learned physiologist commenced his lecture by explaining the nature of the experiment about to be made on the animal. The monkey seemed evidently well pleased by the observations, for he highly amused the whole audience by his mimicries. But when the lecturer went to seize him he cried most piteously, as if he had a presentiment of what was in store for him. Having thrown him on his back and bound him, M. Sequard took the scalpel and was in the act of plunging it into the animal, when, to his great surprise, it was adroitly sent whirling across the room by a straight tip from the umbrella of an indignant lady member of the Anti-Vivisection Society. The humiliation of the lecturer can be easily imagined; however, recovering his *sang froid*, he requested his assailant to leave the hall, but was met with a stern refusal from the lady, who said that as she had no other way to show her disgust for such practices, she had determined to prevent them by the means she had chosen. However, the influence of an *agent de paix* was brought to bear on the member of the gentle sex and she left, protesting all the way. The scalpel was picked up, and now that his defender was gone, poor monkey had to submit *bon gre mal gre* to the operation in question, and the lecture was finished without further interruption.

---

A Methed of Rendering the Skin Insensible in Operations without Chloroform.—M. Jules Guerin read a note at the Academie des Sciences upon a method of rendering the skin insensible in those operations which do not admit of chloroform by inhalation, and cited a case in which he had employed it to advantage. A lady, æt. 60, consulted him three months ago for a tumor of eight years' standing, which, on examination, proved to be a scirrhus. The general health was bad, bronchial and cardiac troubles were very manifest, and the kidneys were not in a very satisfactory condition. However, the operation was urgent. Chloroform having been considered dangerous, M. Guerin applied around the tumor a circular layer of Vienna paste, limited by a double band of diachylon. At the end of twenty minutes the caustic was removed, leaving in its trace a black ribbon-like line. The knife was then applied, and the tumor removed without the patient feeling the slightest pain, and who did not seem to be aware of the operation. The results were all that could be desired.

---

Bright's Disease.—At the Academie de Medecine M. Seminola, of Naples, explained his theory on Bright's disease. According to him, the alterations observed in the renal tissue are not the cause, but the result, of the infiltration of albumen. The disease attacks the liquids before the solids. The albumen is altered in its quantity and composition. Its characters in the serum of the blood are not normal, and consequently it must be eliminated from the economy. M. Seminola injected by little quantities under the skin of healthy animals albumen of different kinds. After some time the kidneys became affected in a similar manner as that observed in Bright's disease. This new theory was tacitly received by the meeting, and immediately afterwards a discussion on the report of the Typhoid Fever Commission was commenced. This Commission was formed to furnish the authorities with advice on the best prophylactic means to be adopted against that affection.

---

Coffee and its Effects.—Dr. P. A. Wilhite read a paper with this title before the last meeting of the South Carolina Medical Association (*Transactions*) in which, after reciting the usual poisonous properties of coffee, he claims that it is more injurious than is generally supposed by the laity or admitted by the profession. He believes that strong coffee, when used to excess, will at least promote, if not cause gout; and its continued use seriously impair sthe blood, irritates the kidneys, congests the liver, deranges the nervous system and paralyzes the digestive functions in all their actions, causing acidity, heartburn, tremor, debility, irritability, dejection of spirits, etc. His attention was directed in an especial manner to these poisonous properties during the late war, when women for whom he had frequently prescribed for a complication of nervous troubles, being unable to procure coffee, became comparatively strong and healthy before the close of the war. He considers that it is not so much the quantity taken, as the peculiar susceptibility of some persons to its influence.

---

The possible value of an intravenous injection of a solution of common salt should not be lost sight of in the emergency which calls for transfusion. This fluid is always available, is safer than the transfusion of blood and requires less skill and simpler apparatus for its use. The only outfit needed is a glass funnel, a few feet of rubber tubing and a canula improvised from a bit of glass tube. The solution should be of six per cent strength, and made with boiled water. It should be rendered slightly alkaline by the addition of a few drops of liquor potassæ, should be filtered and slowly injected at the temperature of 100° F. The volume may be from one to two pints. The writer of this note has found a drachm of liquor ammonia a useful ingredient in this solution.

---

Dr. Webb Kelly, in the *Columbus Medical Journal*, reports a number of cases of carbolic acid poisoning by the constant use of injections of carbolized water by women to prevent conception. He says cases of this kind are of daily occurrence in American practice, where the constant use of this agent produces a mild form of poisoning, great nervous prostration, and irritability of the parts affected Withdrawal from connubial intercourse for a time, and the consequent carbolized injections, in most cases resulted in the re-establishment of health.

---

In the principal foreign cities the rates of mortality per 1,000 of the various populations were, according to the latest official returns, as follows:—Calcutta 36, Bombay 28, Madras, 34, Paris, 27, Geneva 19, Brussels 26, Amsterdam 26, Rotterdam 23, The Hague 21, Copenhagen 21, Stockholm 22, Christiana 13, St. Petersberg 33, Berlin, 27, Hamburg 28, Dresden 25, Breslau 39, Munich 33, Vienna 32, Prague, 25, Buda-Pesth 31, Venice 22, Lisbon 32, New York 29, Brooklyn 20, Philadelphia 21, Baltimore 19.

---

The highest annual death-rates from diseases of the zymotic class in the large towns were—from measles, 1.9 in Liverpool and 2.8 in Newcastle-upon-Tyne; from whooping cough, 1.2 in Manchester and Cardiff, and 1.4 in Plymouth; from scarlet fever, 1.9 in Sheffield and 2.0 in Wolverhampton; and from "fever," 2.0 in Portsmouth. The zymotic death-rate in Glasgow reaches to 8.2 of the entire mortality. The 27 deaths from diphtheria in the large towns included 15 in London and 5 in Glasgow. Small-pox caused 3 deaths in London, 1 in Newcastle upon-Tyne, and one in Hull.

---

THE TREATMENT OF POST PARTUM HEMORRHAGE.—(*Medical Times and Gazette*).—In an address opening a discussion at the Harveian Society, London, Dr. Percy Boulton, Physician to the Samaritan Free Hospital, made the following statements for the prevention and treatment of puerperal hemorrhage:

That a difference in the frequency of the second and third stages of labor exists, which accounts for the management of these cases in the practice of some practitioners. But there are undoubtedly certain *predisposing* causes, such as hemorrhagic diathesis, over-distension of the uterus from multiple pregnancy, excess of liquor annii, uterine tumors and exhaustion from various causes, which no man can avoid.

Firm uterine contraction depends on the nervous supply of the womb. If this has been exhausted by a long and tedious labor, that should have been terminated artificially hours if not days before, what wonder that post-partum hemorrhage to an alarming extent follows uterine inertia?

It must, therefore, be evident that *prevention* of post-partum hemorrhage opens up all the methods of treatment adopted for terminating labor in a reasonable time, before the patient's strength is exhausted.

A relaxed uterus without hemorrhage might make it appear that contraction is not the *sine qua non*. In all such cases the uterine sinuses must have become first close by thrombus. In some anæmic patients this does not occur readily.

The measures to be adopted in the treatment are given in the order that would be followed according to the severity of the case.

The child being born, the patient should be rolled onto her back, the uterus being grasped and firmly held, and the placenta expressed in due time. Not being touched until it has passed the vulva, it should then be removed slowly, turning it over and over, so as to twist the membranes which follow into a cord, while the left hand grasps the uterus.

If the membranes do not follow easily, the cause should be sought. If the placenta and membranes are found to be entire, a dose of ergot should be administered by the mouth and the uterus supported for about one-half hour, after which the patient should be firmly bound. If from any cause the placenta be retained, or does not come away entire, the uterus must be emptied by the hand

Should hemorrhage in the meantime supervene, pressure on the fundus will expel the clots, and friction and kneading of the uterus will excite and keep up uterine contraction. It will be well to inject hypodermically into the thigh or buttock twenty drops of liquid extract of ergot.

The patient's head should be lowered, the room kept cool, and stimulants administered according to the pulse and state of exhaustion.

In mild cases these remedies will usually suffice. Should these fail or the case from the first be one of grave character, other treatment will be necessary.

Troublesome hemorrhage may result from laceration of the cervix uteri or the rup'ure of a vulvæ varix; or from inversion of the uterus. The first two are rarely formidable, while the latter is readily diagnosed and controlled by replacement.

Unfortunately it is only a short step from a simple case of post-partum hemorrhage to one of the gravest that man can possibly have to deal with. These are not cases that allow time to send for assistance, and division of responsibility, but have to be met and dealt with alone without a moment's hesitation. Beyond what has already been mentioned, what are our resources under such an emergency?

The uterus is empty, ergot by the mouth and hypodermically has been given, and in spite of pressure and kneading of the fundus, the organ relapses and serious hemorrhage continues.

Galvanism need scarcely be considered, as a battery is rarely at hand, otherwise it is a most valuable remedy.

Cold, heat, the application of iodide of iron to the uterine lining and transfusion are the remedies which we have to select from.

The injection hypodermically of sulphuric ether, compression of the abdominal aorta, with bandaging of the extremities are supplementary measures.

*Cold*—When ice is at hand it is usual to pass a piece into the vagina, and also to apply it to the hypogastric region, or inject ice cold water into the uterus, applying cloths wrung out in cold water or vinegar and water, to the vulva.

*Heat* is preferable where the state of exhaustion is extreme. Water at 110° or 120° must be injected into the uterus up to the very fundus by means of a syphon syringe. Should these have failed, and life is undoubtedly at stake, add to the hot water one quarter of the bulk of liq. ferri perchlor (not fortior), applied by means of the syringe already in use.

It is not an easy matter to pass the hand grasping a sponge full of fluid through the vagina without losing a great part of the solution. At a time when a man must be calm and have his wits thoroughly about him, it is important not to run any risk of failure, and I commend the syringe as the preferable method.

When enough of blood is lost that fatal syncope is imminent, the bulk of circulating fluid is sufficient to excite the heart to contract, but the blood pressure within the cranium and oxygen-carrying fluid is so reduced that the central sources of nerve energy cease to act.

From recent experiments we know that the heart continues to contract even when removed from the body, so long as the nerve ganglia in its substance are stimulated.

When the patient shows symptoms of impending death, auto-transfusion should be put into practice : Lower the head by elevating the foot of the bed to an angle of 45°; bandage the extremities tightly; put a synapism over the heart. If the patient can still swallow, give a full dose of opium and brandy ; if not, inject hypodermically twenty minims of sulphuric ether.

*Now transfuse.*—Do not hesitate to operate so long as the heart is still beating and the lungs active. Use an instrument which any one can make for himself, composed of two glass canulas, six inches of rubber tubing about one-quarter of an inch in diameter and a pair of spring clips.

From *artery to artery* is far too difficult for anything but laboratory experimentation.

From *vein to vein* is by no means an easy operation, as the veins are empty and hard to find. The apparatus should be put into a cup of salt and water 100° (one table-spoon to the pint) to drive out all the air.

The vein of the donor should be opened, *after exposure by dissection*, a tape put around the arm to stop the flow till wanted, one canula passed into and tied in the vein in the opposite direction to the venous current. A clip should be on the tube close to the other canula, to retain the saline solution. The recipient's arm must next be prepared, and an oblique opening made into it to insert the other canula, first relaxing the tape of the giver's arm and the clip on the tube, so as to fill the apparatus entirely and exclude all air. The transfusion may be allowed to go on, the quantity of blood transfused being determined by the result.

In the absence of human blood a saline solution at 100° is certainly the safest substitute, as it is the easiest to obtain. Common salt used in the proportion of a teaspoonful to a pint of water at 100°. Hot water alone may be injected.

The same canula attached to eighteen inches of tubing with a funnel at the other end. Care must be taken to exclude the air. The solution will pass into the vein by gravitation alone by raising the funnel, the current regulated by compressing the tube.

Whether blood, or a saline mixture, or only hot water is used, it should be a maxim in midwifery that no woman should die of puerperal hemorrhage without being transfused.

---

BISMUTH AS A SPECIFIC FOR CANCRUM ORIS.—*C. J. Macguire, M.D., in N. Y. Med. Record.*—The author had under his care twenty-four cases of this affection, in most of them the disease having followed measles. As to the accuracy of his diagnosis, he is careful to guard himself by giving the testimony of other physicians. Four of his cases were fatal, and those four before he instituted the bismuth treatment, but under this treatment all of the remaining twenty recovered, though many of them were cases apparently hopeless. Such a percentage of recoveries, the author justly remarks, has hitherto not been met with. His method of using bismuth, and the progress of some of the successful cases, may be given in his own words.

On May 11 I was informed, upon my entrance to the ward, that still another child was, in the words of the sister, "getting the frightful disease in the mouth." This child, Katie H., aged seven years, I examined, and found she had had a small ulcer on the inside of the left cheek, with all the characteristics described in the early stages of the other cases. I confess I was in despair. On consideration, I came to the conclusion that following in the old rut of treatment was almost useless, if not quite so. By a process of reasoning, or by accident if you will, I conceived the idea of applying locally the subnitrate of bismuth. After thoroughly cleansing the mouth with a disinfectant lotion, I covered the ulcerated surface with this drug, and the next day, May 12, the grayish slough had partly cleared away, and the fetor, which had been most disagreeable, was sensibly lessened. The hardness of the cheek, if not less, had not increased, and the child was not so thirsty. Temperature, 99° Fahrenheit; pulse, 80. Gum on upper jaw reddish purple, soft and tender. Mouth washed out with solution of carbolic acid; bismuth applied every three hours; syr. ferri iodid., cod-liver oil, and generous diet.

May 13: Fetor markedly less.

May 14: Much better, sleeps well, ulcerated parts getting a healthy appearance. From this date to the first of June, when she was discharged thoroughly cured, the patient did well. On May 12, after witnessing the happy change effected by the bismuth in the case of Katie H., I determined to try its efficacy on Arthur W. and Nellie H., both of whose cases we looked upon as hopeless. On May 10 I had removed some teeth, together with a large piece of the superior maxilla, from Arthur W. His cheek was then swollen tense, a large black gangrenous ulcer was on its inside surface, and his eye was nearly closed. The stench from him was intolerable. Usual disinfecting and supporting treatment continued.

May 11: No change.

May 12: Filled up cavity in cheek with bismuth, first cleansing it out well with disinfectant wash, and repeated every three hours.

May 13: No marked change, except odor a little lessened.

May 14: No increase of gangrenous erosion. Treatment continued. Separation of a large mass of grayish black slough from ulcer on cheek and cavity whence exfoliated bone was removed. Parts looking healthier, fetor less.

May 18: Fetor nearly disappeared in toto! Takes food freely; sleeps better, ulcer assuming a very healthy appearance; swelling and hardness of the tissues of the cheek rapidly subsiding.

May 25: Ulcer on inside of cheek quite filled out and granulating kindly. Removed small piece of dead bone from superior maxilla. Otherwise doing well. Treatment continued. From this date to July 1, when discharged cured, the patient did excellently well. Since then he has been taking cod liver oil, and is now in perfect health. Nellie H. ran the same course, up to May 12, as the two preceding. Her condition at that time was identically the same—black gangrenous slough on buccal mucous membrane, swelled cheek,t etc. Applied treatment as in the last case.

May 13: Clipped away with scissors a considerable amount of disintegrated tissue, then packed parts with bismuth. Continued treatment to 17, when I removed a large piece of exfoliated bone and several teeth from inferior maxilla. From this date to June 15 she slowly but steadily improved, the bismuth being regularly applied, and the mouth thoroughly syringed. Iron, cod-liver oil, and generous stimulating diet administered. She is now one of the healthiest children in the institution, though showing the loss of hard and soft tissue, but *not* showing the horrible deformity which formerly cases that recovered under ordinary treatment suffered from.

---

An Ethical Incident.—*By Owen D. Pomeroy, in N. Y. Medical Journal.*—Sir: A few weeks since I was asked by some of my friends to meet a medical man in consultation who was described as being a very liberal homœopathist. I naturally hesitated, as I was fearful that no argument of opinion might be reached, and, consequently, I, however, made an appointment, and on meeting the medical man in question, I propounded some questions to him in order to find whether we could meet on any common ground. To my great surprise, I found him to be a graduate of the University of the City of New York, a very intelligent man, and apparently well trained in the healing art.

We proceeded with the consultation; there was nothing peculiar or heterodox about his opinion, he responded promptly to my suggestions, everything went on smoothly, and the patient made a good recovery. In a subsequent conversation I taxed him with the question, Why did he call himself a homœopathic physician? He answered that some years since a neighboring physician had reported him to Dr. ——, who had been called the drill sergeant of the profession, as having infringed the rules by using pulsatilla in his practice. This was not denied, the doctor stating that he regarded it as a good remedy, and that he intended to continue its use. For this offense his name was removed from our medical register. The doctor stated to me that he then felt that he must belong to some medical organization, so he joined a homœopathic society. He at this juncture, however, affirmed, with almost an air of ferocity, "But I am none of your little pill men. He expressed a disgust for some of his associates who were infinitismalists, at the same time remarking that others, however, were good physicians in the best sense of the term, not having any dogmas, but who selected remedies just as we do—to wit: the one, in our knowledge and experience, as being the best adapted to a given case. He expressed a determination, which has been since acted upon, to resign from the society. I have told him that, if he desired it, I would make an effort to cause him to join our county society.

He proposes to do so after a little time. This has caused me to reflect upon the fact that there are many brethren in the homœopathic ranks who are essentially with us, and who deserve to sit in our councils. Webster defines a physician as being "a person skilled in the art of healing." Do we doubt that many of our homœopathic friends are otherwise than of this sort? Can we consistently assert that all these men are quacks, imposters and ignorant men?

If we cannot, then it surely follows that the attitude of the profession, as shown in the application of the old code, violates the law of equity, certainly of charity, and is at variance with the teachings of any perfect code which regulates the conduct of one gentleman toward another.

---

MASTURBATION AS AN ETIOLOGICAL FACTOR IN THE PRODUCTION OF GYNIC DISEASES —Dr. Chapman (*American Journal of Obstetrics*) closes a thorough and valuable paper on the above subject with the following conclusions:

(1) That masturbation exists to a considerable extent in women.

(2) That it is accomplished, as a rule, by manipulation of the external parts

(3) That its accomplishment by the introduction of foreign bodies into the vagina is in this country (Scotland) rare.

(4) That decided and constant changes of the external organs of generation result from its long-continued practice.

(5) That the presence of such changes (which are fully described in the paper) is sufficient to warrant the assumption of the existence of the habit.

(6) That by judicious questioning and the avoidance of making accusations of moral obliquity to such patients, this assumption may be strengthened and in many cases confirmed.

(7) That, apart from the use of any mechanical dilating means, masturbation is capable of producing very marked relaxation of the vagina.

(8) That retroversion of the uterus is of such common occurrence among masturbators that its existence in an unmarried nullipara should always be regarded with extreme suspicion.

(9) That the same suspicion should be shown on the occurrence of leucorrhœa and menorrhagia *together* in similar patients.

(10) That ovarian pain and even chronic ovaritis may be set up by the habit

(11) That many other affections of the female generative organs may also be thus occasioned.

(12) That masturbation has so frequently a distinct etiological connection with disease of the pelvic organs, that its recognition will often prove a valuable aid toward forming a prognosis in, and directing a line of treatment for, many uterine complaints in the unmarried.

---

Iodoform in Diphtheria.—E. Sesemann (*Petersb. Med. Wochenschrift*) has used iodoform in the local treatment of diphtheria. He applies it mixed with three parts of sugar with a powder-blower. He considers it necessary that the powder should reach the posterior face of the soft palate and the mucous membrane of the nose. In order to be certain of this, a flexible tube is introduced through the nose and the powder thrown through this, or the iodoform is made into little rolls with vaseline and these, pushed back into the posterior nares, are allowed to dissolve.

Before the powder is applied with the blower, older children should cleanse the fauces by gargling a solution of borax, and the same solution should be thrown as a spray into the throats of younger children.

In severe cases the powder should be applied every hour or two. The accidental swallowing of the iodoform will do no harm. Of twenty one severe cases thus treated, eighteen of the children being under six years of age, five died (23.8 per cent).

Dr. Korach (*Wien. Med. Blatter*) has also used iodoform quite extensively in the treatment of diphtheria. He employs starch containing two per cent. of iodoform six times daily; or he applies a solution of iodoform in collodion or ether. Out of one hundred and twelve cases so treated, seventy two being light and forty severe, eight died.

---

Dr. James M. Holloway, Prof. General and Clinical Surgery, Hospital College of Medicine, Louisville, Ky., says: "I have administered this hypnotic almost daily for many months. When diluted with ice-water it is not objectionable to those who are suffering from indigestions that accompany the advanced stages of phthisis and other exhaustible diseases. I am convinced by careful tests that it is one of the best soporifics. It is not followed by the depression that oftentimes renders the preparations of opium and other narcotics so objectionable. Bromidia seems to be uniform in strength and effect."

---

An esteemed Chicago acquaintance sends us the following: "A physician living on the South Side was recently called in to catheterize the bladder of a man said to be dying from kidney disease and suffering from retention. On his arrival he found the patient comatose, and in the care of two female physicians of the homœopathic persuasion, one of whom had been vainly endeavoring to reach the bladder with a female catheter, which she tendered him on his arrival, alleging as the cause of her failure that she was not accustomed to catheterize the male bladder. Saying that he had an instrument of his own, which he was used to, he drew off twenty-four fluid ounces, and retired with a firm convinction that this was one of those events which make one smile even in the presence of death."

---

The Dangers of Illicit Drinking.—Nothing can show more conclusively the necessity for vigilance on the part of the authorities in suppressing illicit drinking than an analysis made of five samples of Greenock shebeen whiskies. Two were simply diluted Berlin spirit; a third was a raw grain whisky containing about one per cent. fusel-oil, and three to four per cent. methylated spirit; the fourth was an inferior whisky, containing six per cent. of methylated spirit and artificial flavoring matters; while the fifth was a mixture of malt and Berlin spirit with about forty-three grains of vitriol.

---

I HAVE used CELERINA in several cases of general nervous debility in female convicts at the City Work House, and can say from the results in these cases I consider it surpasses Bromides and Hydrate Chloral. I can cheerfully recommend it to the medical profession, and will continue its use at City Work House. M. K. ALLEN, M.D.,

Louisville, Ky. Physician in Charge City Work House.

---

A WRITER in the *Medical Brief* says that flowers of sulphur, one drachm; petrolium mass, one ounce, mixed, will cure pruritus ani every time if applied night and morning. Cosmoline, vaseline or lard will not do with the sulphur.

